# Cytomegalovirus Immune Responses and Cognitive Impairment in People With Human Immunodeficiency Virus

**DOI:** 10.1093/infdis/jiag239

**Published:** 2026-04-28

**Authors:** Lakshmi Chauhan, Debashis Ghosh, Beau Ances, Katherine Tassiopoulos, Kunling Wu, Adriana Weinberg, Kristine M Erlandson

**Affiliations:** Department of Medicine, University of Colorado Anschutz Medical Campus, Aurora, Colorado, USA; Department of Biostatistics, Colorado School of Public Health, University of Colorado Anschutz Medical Campus, Aurora, Colorado, USA; Department of Neurology, Washington University, St Louis, Missouri, USA; Center for Biostatistics in AIDS Research, Harvard T.H. Chan School of Public Health, Boston, Massachusetts, USA; Center for Biostatistics in AIDS Research, Harvard T.H. Chan School of Public Health, Boston, Massachusetts, USA; Department of Pediatrics, University of Colorado Anschutz Medical Campus, Aurora, Colorado, USA; Department of Medicine, University of Colorado Anschutz Medical Campus, Aurora, Colorado, USA

**Keywords:** cognitive impairment, CMV immune response, ELISpot, CMV IgG, frailty

## Abstract

People with human immunodeficiency virus (HIV) have an earlier occurrence of aging complications, including cognitive impairment (CI). Chronic immune activation from cytomegalovirus (CMV) may contribute to increased inflammation and CI. We explored this association using several measures of CMV immune response among people with HIV (PWH). In a case-control design, we compared CMV immunoglobulin G (IgG), enzyme-linked immunospot (ELISpot), and flow cytometry between cases (subsequent CI) and controls (no CI). Baseline CMV IgG, CMV ELISpot, or flow measures were not associated with subsequent CI. This exploratory analysis supports larger studies using more nuanced immunophenotyping to clarify relationship between CMV and development of CI in PWH.

## Teaser

Cytomegalovirus (CMV) IgG and ELISpot were not significantly associated with cognitive impairment in people with HIV. Small differences in phenotypic and functional flow suggest that larger studies using nuanced immunophenotyping are needed to clarify relationships between cognitive impairment and CMV.

People with human immunodeficiency virus-1 (HIV) now have a near-normal life expectancy, resulting in more than 50% of people with HIV (PWH) aged >50 years. PWH continue to have excess morbidity and mortality including an earlier occurrence of aging complications such as dementia [1, 2]. Although the incidence of HIV-associated dementia has declined with HIV suppression, the prevalence of milder cognitive impairment (CI) and age-associated dementias have remained stable or even increased [3]. Cerebrovascular disease risk factors (hypertension, diabetes), psychiatric comorbidities, viral coinfection, and substance use disorders can contribute to CI along with direct effects of HIV.

Chronic immune activation from latent infections such as cytomegalovirus (CMV) may have a role in chronic diseases, including neurodegenerative disorders [4, 5]. CMV seropositivity and high proportions of terminally differentiated CMV-specific T cells characterize an “immune risk profile” that is associated with increased mortality risk among elderly populations [6]. Increased proportion of CMV-specific CD4^+^ T cells and higher CMV IgG have been linked to neurofibrillary tangles and Alzheimer disease neuropathology [5]. Among PWH, CMV immunoglobulin G (IgG) or other CMV-specific CD4^+^ or CD8^+^ T cells have been associated with cardiovascular disease, frailty, and CI [4, 7, 8].

In this study, we leveraged a longitudinal observational cohort with stored samples and measures of cognitive function to evaluate the association between CMV antibody and cell-mediated responses and subsequent CI among PWH.

## MATERIALS AND METHODS

### Study Participants

Participants were selected from the observational study Advancing Clinical Therapeutics Globally for Infectious Diseases (ACTG) ALLRT (A5001, enrolled 2000–2011, followed through 2013); a subset of ALLRT participants (those 40 and older) were also enrolled and followed into another observational cohort, HAILO (A5322, enrolled 2013–2014, followed through 2021) at completion of ALLRT [9]. All participants for this analysis were antiretroviral therapy (ART) naive upon enrollment into ALLRT. Both the ALLRT and HAILO studies obtained institutional review board approval at the individual study sites, and all participants signed written informed consent prior to enrollment.

### Study Design

We conducted a case-control study among virally suppressed participants (2 consecutive HIV-1 RNA <50 copies/mL) who had ≥6 months of ART at baseline and no viral blips (≥200 copies/mL) through follow-up. Cases had no CI at baseline but developed CI during follow-up. Controls had no CI at baseline and throughout follow-up. Cases and controls were matched on CD4 category (<200 vs ≥200 cells/µL), sex, age, and years of follow-up. We identified 48 case-control pairs with clinical data and specimens at both time points. CMV immunity measures were performed at baseline and follow-up.

For CI assessment, NPZ-3 scores were calculated every 48 weeks as the average Z-score of 3 tests: Trail Making Tests A and B and the Wechsler Adult Intelligence Scale–Revised Digit Symbol Test. Raw component scores were adjusted for demographic factors (sex, age, race, educational status, learning effects) using normative data. Positive scores indicated better neurological function, and negative scores indicated worse neurological function. CI was defined by ≥1 standard deviation (SD) below the mean on 2 tests or ≥2 SD below the mean on 1 test on the NPZ-3.

### CMV Immunity Measures

#### CMV IgG

Plasma was thawed overnight at 4°C and tested using a CMV IgG Kit (Gold Standard Diagnostics; 01-140) according to manufacturer's protocol, with modifications for semi-quantitative analysis. A standard curve was built using 3-fold serial dilutions of a known CMV-seropositive plasma sample included on each plate. After blank subtraction, optical density values were interpolated onto the standard curve of the quantitative control using log-transformed nonlinear fit algorithm and GraphPad Prism software version 10.4.0 for Windows. Concentrations were reported in enzyme-linked immunosorbent assay (ELISA) units (EU/mL).

#### Enzyme-Linked Immunospot Assay

Cryopreserved peripheral blood mononuclear cells (PBMCs) were thawed and rested overnight at 37°C and 5% CO_2_ at 10^6^ PBMCs/mL in growth medium consisting of RPMI 1640 with L-glutamine (Gemini BioProducts), 10% human AB serum (Gemini BioProducts), 2% HEPES buffer (Mediatech), and 1% penicillin-streptomycin (Gemini BioProducts). The next day, 96-well Millipore enzyme-linked immunospot (ELISpot) plates (Sigma) were coated with anti-human interferon gamma (IFN-γ) antibody (Mabtech, mAb1-D1k). Rested PBMCs were added at 250 000 cells/well in duplicate wells and stimulated for 20 hours at 37°C in a humidified atmosphere and 5% CO_2_ with UV-irradiated CMV-infected cell lysate, mock-infected control, and phytohaemagglutinin. A study-specific leucopack PBMC sample with known CMV reactivity was included in each run for assay validation. Plates were incubated with secondary anti-human IFN-γ conjugated with (Mabtech, mAb 7-B6-1) and developed with the colorimetric substrate 5-bromo-4-chloro-3-indolyl phosphate/nitro blue tetrazolium (Mabtech). Results were reported as the mean number of spot-forming cells (SFCs)/10^6^ PBMCs in CMV-stimulated wells after subtraction of SFCs in mock-stimulated wells.

#### Phenotypic and Functional Flow Cytometry Analyses

Please see detailed methods in the Supplementary Materials. All flow cytometric analysis was performed on FlowJo software (BD Biosciences). We used the absence of CD8 expression to identify CD4^+^ T cells and applied the CD8^+^ gating strategy to γδ T cells, as these cells may express CD4 constitutively or following activation (Supplementary Figure). In stimulated flow, we specifically evaluated expression of CMV-protective phenotypes (NK^+^IFN-γ^+^, NK^+^CD57^+^, NK^+^PD-1^+^, CD8^−^ γδ IFN-γ^+^) and cells associated with CMV end-organ disease (CD4^+^CD25^+^FoxP3^+^ and CD4^+^PD-1^+^).

### Statistical Analysis

We compared cases and controls using descriptive statistics. Conditional logistic regression was used to determine associations between CMV IgG and case/control status, and adjusted for age, race, sex, education, baseline CD4 count, baseline HIV-1 RNA, and neuropathy. Logistic regression analysis was used for CMV ELISpot due to missing viable paired samples. For our exploratory aim, we descriptively compared phenotypic and stimulated flow cytometry in cases and controls with a focus on the markers that have been previously associated with protection against CMV end-organ disease in the literature. We used 2-sided tests with a significance level of .05. Descriptive statistics were performed for phenotypic and stimulated flow cytometry. Analyses were conducted in R version 4.4.1 software.

## RESULTS

We included 48 cases and 45 controls for the primary CMV IgG ELISA, 23 and 29 for ELISpot, 15 and 17 for phenotypic flow cytometry, and 14 and 15 for functional flow cytometry based on sample availability and viability. Cases and controls had similar ages (42.6 and 42.3 years, respectively), baseline CD4 count, and HIV RNA copies/mL of plasma (Table 1). Neuropathy was present in more cases (35.4%) than controls (17.8%). Baseline median NPZ-3 scores were lower in cases (−0.350) than controls (0.733). The mean duration between baseline and follow-up was 6.5 (SD, 3.4) years.

**Table 1.** Demographic Characteristics of Cases and Controls at Baseline

Characteristic	Cases (n = 48)	Controls (n = 45)	Overall (N = 93)
Age, y	42.6 (9.6)	42.3 (8.0)	42.5 (8.8)
Sex			
Male	40 (83.3%)	37 (82.2%)	77 (82.8%)
Female	8 (16.7%)	8 (17.8%)	16 (17.2%)
Race/Ethnicity			
Hispanic/Latino	10 (20.8%)	3 (6.7%)	13 (14.0%)
Black	9 (18.8%)	19 (42.2%)	28 (30.1%)
White	29 (60.4%)	23 (51.1%)	52 (55.9%)
Educational status, y	13.9 (3.6)	14.7 (3.0)	14.3 (3.3)
Body mass index, kg/m^2^	27.1 (5.9)	27.0 (3.5)	27.0 (4.8)
Baseline HIV RNA, copies/mL	47.3 (3.8)	46.8 (4.2)	47.1 (4.0)
Baseline CD4 T-cell count, cells/μL	477 (234)	453 (213)	465 (223)
CD4 T-cell count at study entry^a^, cells/μL	272 (210)	225 (175)	…
Hepatitis C virus infection	3 (6.3%)	4 (8.9%)	7 (7.5%)
Hypertension	12 (25%)	17 (37.8%)	29 (31.2%)
Diabetes	3 (6.3%)	3 (6.7%)	6 (6.5%)
Cardiovascular disease	0 (0%)	0 (0%)	0 (0%)
Renal disease	3 (6.3%)	2 (4.4%)	5 (5.4%)
Neuropathy	17 (35.4%)	8 (17.8%)	25 (26.9%)
Psychiatric disease	2 (4.2%)	0 (0%)	2 (2.2%)
Statin use	4 (8.3%)	2 (4.4%)	6 (6.5%)
NPZ-3^b^ score, median [min, max]			
Baseline	−0.350 [−0.967, 1.10]	0.733 [−0.667, 2.17]	0.200 [−0.967, 2.17]
Follow-up	−1.05 [−3.07, −0.37]	1.17 [−0.30, 2.73]	−0.367 [−3.07, 2.73]

Values are presented as mean (standard deviation) or number (%) unless otherwise indicated.

Abbreviation: HIV, human immunodeficiency virus.

^a^As participants started antiretrovirals at or near study entry, the CD4 count at study entry is an approximate for CD4 nadir.

^b^NPZ-3 is the neuropsychological composite score from Trail Making Tests (series A and B) and the Digit Symbol Test (Wechsler Adult Intelligence Scale–Revised). NPZ-3 is the sum of 3 test scores standardized for age.

Three samples were seronegative for IgG: 2 of these were excluded from ELISpot due to low cell viability; all 3 were excluded from flow cytometry.

### Baseline and Change in CMV IgG

At baseline, mean CMV IgG was similar between cases (9.57 [SD, 6.37]) EU/mL and controls (9.37 [SD, 6.27]) EU/mL. The odds of CI was not significantly associated with baseline CMV IgG in unadjusted (odds ratio [OR], 0.94 [95% confidence interval, 0.59–1.48]) or adjusted analyses (OR, 1.07 [95% confidence interval, 0.61–1.92]). Follow-up CMV IgG was also similar between cases (8.47 [SD, 5.61]) and controls (8.78 [SD, 5.58]) and decreased in both cases (−1.10 [SD, 3.72]) and controls (−0.59 [SD, 6.19]).

### Baseline and Change in CMV ELISpot

Mean baseline ELISpot was 90.94 (SD, 65.70) SFCs/10^6^ PBMCs among cases (n = 26) and 108.54 SFCs/10^6^ PBMCs (SD, 62.04) among controls (n = 31) (Table 2). The odds of subsequent CI was not associated with baseline ELISpot in unadjusted (OR, 0.99 [95% confidence interval, 0.98–1.00]) or adjusted analyses (OR, 1.00 [95% confidence interval, 0.98–1.01]). On follow-up, the mean ELISpot increased in cases (n = 36) to 111.89 (SD, 70.69) SFCs/10^6^ PBMCs and in controls (n = 40) to 131.97 (SD, 64.29) SFCs/10^6^ PBMCs. Correlations between CMV ELISpot and CMV IgG at baseline and follow-up were weak and not significant (*r* = 0.07 and *r* = 0.02; both *P* >0.2).

**Table 2.** Immune Measures at Baseline and Follow-up in Cases and Controls

Immune Measure	Cases		Controls	
	Baseline	Follow-up	Baseline	Follow-up
CMV IgG, EU/mL	9.57 (6.37) n = 48	8.47 (5.61) n = 48	9.37 (6.27) n = 45	8.78 (5.58) n = 45
ELISpot, SFCs/10^6^ PBMCs	90.94 (65.70) n = 26	111.89 (70.69) n = 36	108.54 (62.04) n = 31	131.97 (64.29) n = 40
Functional phenotypic flow cytometry analysis^a^	n = 15	…	n = 17	…
Monocytes CD83^+^	9.58 (4.56)	…	9.88 (5.83)	…
Monocytes PD-L1^+^	0.13 (0.13)	…	0.11(0.08)	…
Monocytes intermediate	2.88 (2.94)	…	3.30 (5.57)	…
Monocytes nonclassical	1.79 (1.90)	…	1.33 (0.67)	…
Monocytes CD16^+^	4.62 (4.58)	…	4.57 (5.78)	…
NK cells NKG-2C^+^CD57^+^	3.17 (4.28)	…	4.08 (5.48)	…
NK cells CD57^+^	41.16 (23.20)	…	32.73 (15.07)	…
NK cells NKG-2C^+^	9.74 (10.57)	…	13.89 (13.48)	…
T cells CD4^+b^CD38^+^HLA-DR^+^	7.10 (6.22)	…	6.04 (3.35)	…
T cells CD4^+^CD57^+^CD28^−^	17.86 (13.20)	…	19.97 (10.57)	…
T cells CD4^+^PD-1^+^	9.79 (4.37)	…	9.88 (3.77)	…
T cells CD4^+^TIM3^+^	0.57 (0.48)	…	0.58 (0.46)	…
T cells CD8^+^CD38^+^HLA-DR^+^	12.13 (7.77)	…	9.43 (5.25)	…
T cells CD8^+^CD57^+^CD28^−^	37.20 (12.54)	…	39.53 (17.96)	…
CD8^+^PD-1^+^	6.53 (3.85)	…	6.61 (3.28)	…
CD8^+^TIM3^+^	0.68 (0.53)	…	1.05 (1.01)	…
CMV-specific^c^ flow cytometry analysis	n = 14	…	n = 15	…
NK cells IFN-γ^+^	3.36 (3.40)	…	6.92 (6.84)	…
NK cells CD57^+^	0.34 (3.73)	…	1.30 (3.28)	…
NK cells PD-1^+^	−0.04 (0.15)	…	0.12 (0.26)	…
T cells CD8^−^ γδ^+^ IFN-γ^+^	0.51 (0.94)	…	0.06 (1.32)	…
T cells γδ^−d^ CD4^+^CD25^+^FoxP3^+^	2.59 (2.21)	…	1.39 (0.90)	…
T cells γδ^−^ CD4^+^PD1^+^	0.98 (0.68)	…	0.60 (0.57)	…
T cells γδ^−^ CD8^+^IFN-γ^+^	0.26 (0.22)	…	0.44 (0.46)	…

Values represent mean (standard deviation).

Abbreviations: CMV, cytomegalovirus; ELISpot, enzyme-linked immunospot; IgG, immunoglobulin G; NK, natural killer; PBMC, peripheral blood mononuclear cell; SFC, spot-forming cell.

^a^Functional phenotypic analysis and stimulated flow analysis only measured at baseline.

^b^CD4^+^ T cells represent CD3^+^CD8^−^ T cells.

^c^CMV specificity was defined by CMV-stimulated results after subtraction of mock-stimulated results.

^d^Conventional T cells typically represent >90% of the γδ^−^ T cells, which allowed us to separate the T cells in γδ^+^ and conventional based on presence or absence of γδ TCR (see gating strategy in Supplementary Figure 1).

### Factors Associated With Change in Cognitive Function Between Timepoints

Conditional regression analysis showed that baseline IgG or change in IgG was not associated with subsequent change in cognitive function (*P* = .27 and *P* = .32, respectively; Supplementary Table 1). Presence of neuropathy (*P* = .03), White race (*P* = .03), and Hispanic ethnicity (*P* = .02) showed a significant association (Supplementary Table 1). Baseline ELISpot or change in ELISpot was not associated with subsequent change in cognitive function on logistic regression analysis (*P* = .55 and *P* = .43, respectively; Supplementary Table 2).

### Flow Cytometry Analysis

Phenotypic and functional characteristics at baseline for cases and controls are shown in Table 2. In stimulated flow analyses, expression of NK^+^IFN-γ^+^, NK^+^CD57^+^, NK^+^PD-1^+^, and CD8^+^IFN-γ^+^ conventional T cells tended to be lower among cases, while expression of CD8^−^ γδ IFN-γ^+^, CD4^+^CD25^+^FoxP3^+^, and CD4^+^PD-1^+^ tended to be higher among cases.

## DISCUSSION

In this case-control study of PWH, we found no significant differences in CMV-specific IgG responses or CMV ELISpot at baseline or follow-up among people who did or did not develop CI. Additionally, we did not find correlations between anti-CMV antibodies and ELISpot results. To our knowledge, this is the first longitudinal study to evaluate the association between CMV immune response and CI in PWH. Some key aspects distinguish our study from prior research: (1) All participants were virally suppressed for HIV, (2) we compared immune response before and after development of CI with a median time of 6.5 years, and (3) we evaluated both antibody and cell-mediated responses longitudinally.

The relationship between CMV IgG and CI across different study populations has been inconsistent [10]. CMV antibody concentrations (IgG) may not be a sensitive measure of CMV reactivations. High concentrations of CMV IgG antibodies may correlate with immune responses rather than reactivations [11]. We noted a decrease in CMV IgG levels over time in both cases and controls. In contrast to cross-sectional studies showing associations between CMV IgG and CI, we did not find correlation of CMV IgG levels with subsequent CI [8, 12].

ELISpot, a standardized test used to measure CMV cell-mediated immunity, has been associated with CMV end-organ disease and CI [12]. Although we noted an increase in ELISpot levels with time in cases and controls (likely representing asymptomatic reactivations), there was no association between baseline or change in ELISpot and subsequent CI.

Protection against clinically significant CMV reactivation is primarily attributed to cell-mediated immunity (IFN-γ^+^ and PD1^+^ NK cells), with emerging role of γδ T cells, which undergo clonal expansion and assist with CMV viral clearance [13, 14]. In our exploratory study, these protective phenotypes (NK cells expressing IFN-γ, CD57, and PD-1) tended to be lower in cases. Conversely, CMV-specific Treg (CD4^+^CD25^+^FoxP3^+^) and PD-1–expressing CD4^+^ T cells, which have been associated with CMV end-organ disease, were higher in cases [15]. Interestingly, protective γδ T cells tended to be higher in those who developed CI. Notably, these differences were small, and tests for statistical significance were not performed.

Strengths of this study include baseline and follow-up samples to assess CMV immune responses in a well-characterized cohort with regular cognitive assessments.

Limitations include small sample size and nonviability of some PBMC samples. Cognitive tests used in this cohort (Trail Making and Digit Symbol Coding) reflect executive-motor efficiency or psychomotor functions; other cognitive domains are not well-evaluated.

In summary, although baseline and follow-up CMV IgG and ELISpot were not significantly associated with CI development in PWH, our exploratory analysis supports larger studies using more nuanced immunophenotyping to clarify relationship between CI and CMV.

## Supplementary Material

jiag239_Supplementary_Data

## Notes

***Acknowledgments*.** We acknowledge the contributions of Krystle Garth, who performed ELISpot and flow cytometry analysis. We would like to thank all the participants in this study.

***Data availability*.** De-identified, individual participant-level data that underlies the reported results will be made available upon request after approval of a proposal or with a signed data access agreement (https://actgnetwork.org/submit-a-proposal-2).

***Financial support*.** This work was supported by the National Institutes of Health through the National Institute of Allergy and Infectious Diseases (grant number AI068636) and the National Institute on Aging (grant numbers K24 AG082527 and R01AG054366 to K. M. E.).

## Supplementary Data

Supplementary materials are available at *The Journal of Infectious Diseases* online (http://jid.oxfordjournals.org/). Supplementary materials consist of data provided by the author that are published to benefit the reader. The posted materials are not copyedited. The contents of all supplementary data are the sole responsibility of the authors. Questions or messages regarding errors should be addressed to the author.
