# Supplementary material for: Cytomegalovirus Immune Responses and Cognitive Impairment in People With Human Immunodeficiency Virus
Source: J Infect Dis. 2026 Apr 28;234(3):e461–5. doi: 10.1093/infdis/jiag239 (PMC13570583; doi:10.1093/infdis/jiag239)
Supplement: Supplementary file 1 [file jiag239_supplementary_data.zip › Supplementary Tables.docx]

**Supplementary Methods:**

*Phenotypic flow cytometry* *analysis*

Cryopreserved PBMCs were thawed and resuspended in growth medium, then plated at 250,000–500,000 cells/well in 96‑well polypropylene plates and stained with Zombie Yellow viability dye and monoclonal antibodies against the following surface markers: TIM3 (FITC), CD8 (PerCP-Cy5.5) , CD38 (APC), CD3 (Alexa Fluor 700), CD83 (APC-Cy7), HLA-DR (BV421), CD28 (BV510), CD19 & CD20 (BV605), CD14 (BV650) , PD-L1 (BV711), PD1 (BV785) , NKG-2C (PE), CD16 (PE-CF594), CD56 (PE-Cy5), and CD37 (PE-Cy7). Brilliant Stain Buffer and Monocyte Block were added to the surface‑stain cocktail at 10 µL/well and 2.5 µL/well. Control leucopack sample was included in each run for validation. Agilent Quanteon flow cytometer was used to acquire ≥250,000 PBMC events.

*Functional flow cytometry analysis*

Cryopreserved PBMCs were thawed, resuspended, plated at 250,000 cells/well in 96‑well polypropylene plates, and stimulated overnight with UV‑irradiated CMV‑infected cell lysate or mock control, together with anti‑CD28 and anti‑CD49d monoclonal antibodies (1 µg/mL), at 37 °C and 5% CO₂. Control PBMC sample with CMV reactivity was included for assay validation. Brefeldin A (Sigma B7651) at 5µg/mL was added to each well for the last 4 hours of incubation. Samples were stained with Zombie Yellow and monoclonal antibodies against the following fluorophore‑conjugated surface markers: TIM3 (FITC), CD8 (PerCP-5.5), CD16 (RB780), CD28 (BV510), CD3 (Ax700), TCR γδ (BV605), PD1 (BV785), CD25 (BV650), CD57 (PE-CF594), CD56 (PE-Cy5). Brilliant stain buffer and Monocyte block were used as above. FOXP3 Fixation/Permeabilization working solution and buffers were added to each well. Cells were stained with monoclonal antibodies against intracellular/intranuclear molecules: TGFβ (APC), IFNγ (APC-Cy7), FOXP3 (PE-Cy7), Ki-67 (BV421), IL-10 (BV711), TNFα (PE).

**Supplementary Table 1: Conditional Logistic Regression of Baseline IgG, Change in IgG levels, and other Clinical Demographic Variables on Case-Control Status***

| *Predictors* | *Estimates*  *(95% Confidence Interval)* | *P-value* |
| --- | --- | --- |
| Baseline IgG (EU/mL) | 0.08 (-0.06 – 0.21) | 0.27 |
| Change in IgG (EU/mL) | 0.08( -0.07 – 0.23) | 0.32 |
| Ethnicity (Hispanic) | 3.47 (0.59 – 6.34) | **0.02** |
| Race (White Non-Hispanic) | 2.14 (0.19 – 4.10) | **0.03** |
| Baseline CD4 (cells/mm^3^) | 0 (-0.00 – 0.00) | 0.71 |
| Baseline Age (years) | -0.08 (-0.34 – 0.17) | 0.52 |
| Baseline BMI (kg/m^2^) | 0.01 (-0.12 – 0.14) | 0.87 |
| Education (years) | 0.0 (-0.24 – 0.25) | 0.98 |
| Time between measurements (years) | -0.9 (-2.76 – 0.96) | 0.34 |
| Hypertension | -0.47 (-1.67 – 0.72) | 0.44 |
| Presence of Neuropathy | 1.67 (0.16 – 3.18) | **0.03** |

*Adjusted for confounding factors (age, race, sex, education, baseline CD4 count, baseline HIV-1 RNA, presence of neuropathy

**Supplementary Table 2: Logistic regression of Change in ELIspot levels and other Clinical/Demographic Variables on Case-Control status***

| *Predictors* | *Odds Ratio* | *95% Confidence Interval* | *P-value* |
| --- | --- | --- | --- |
| ELISpot baseline (SFC/10^6^ PBMC) | 1.00 | 0.98-1.01 | 0.55 |
| ELISpot Change (SFC/10^6^ PBMC) | 0.99 | 0.98 – 1.01 | 0.43 |
| Ethnicity (Hispanic) | 1.68 | 0.13 – 23.75 | 0.69 |
| Race (White Non-Hispanic) | 4.43 | 0.84 – 29.33 | 0.095 |
| Baseline CD4 (cells/mm^3^) | 1.00 | 1.00 – 1.00 | 0.87 |
| Baseline age (years) | 1.00 | 0.91 – 1.09 | 0.94 |
| Baseline BMI (kg/m^2^) | 1.02 | 0.88 – 1.17 | 0.79 |
| Male sex | 1.31 | 0.24 – 9.04 | 0.76 |
| Education (years) | 0.78 | 0.53 – 1.09 | 0.16 |
| Time between measurements (years) | 0.86 | 0.65 – 1.11 | 0.27 |
| Hypertension | 1.06 | 0.20 – 5.63 | 0.94 |
| Presence of neuropathy | 4.09 | 0.67 – 31.61 | 0.14 |

*Adjusted for confounding factors (age, race, sex, education, baseline CD4 count, baseline HIV-1 RNA, presence of neuropathy
